# AMPK activation-dependent autophagy compromises oleanolic acid-induced cytotoxicity in human bladder cancer cells

**DOI:** 10.18632/oncotarget.18980

**Published:** 2017-07-04

**Authors:** Yarong Song, Peng Zhang, Yadong Sun, Xuechao Li, Lifeng Chen, Yajun Xiao, Yifei Xing

**Affiliations:** ^1^ Department of Urology, Union Hospital, Tongji Medical College, Huazhong University of Science and Technology, Wuhan 430022, China

**Keywords:** bladder cancer, oleanolic acid, autophagy, AMPK, cytotoxicity

## Abstract

Autophagy is an evolutionarily conserved catabolic process in eukaryotic cells, which allows cells to overcome a wide array of of stresses and has recently been shown to result in drug resistance. This study examined the effect of autophagy on oleanolic acid (OA)-induced cytotoxicity against bladder cancer cells. Our study demonstrated that OA inhibited cell viability, proliferation, and induced apoptosis in bladder cancer lines T24 and EJ. Furthermore, OA induced autophagy in both cell lines by activating AMP-activated protein kinase (AMPK), inhibiting mechanistic target of rapamycin (mTOR) and promoting unc-51 like autophagy activating kinase 1 (ULK1). Moreover, inhibiting autophagy by siRNA to autophagy related 7 (ATG7) or with autophagy inhibitor bafilomycin A1 and 3-methyladenine (3-MA) or AMPK inhibitor dorsomorphin (compound C) promoted OA-induced deaths of bladder cancer cells. In contrast, either autophagy activator rapamycin or AMPK activator acadesine (AICAR) compromised OA-induced anti-cancer effect. Our findings suggested that OA induced protective autophagy through AMPK-mTOR-ULK1 signaling pathway in bladder cancer cells and OA in combination with autophagy inhibitor might be a novel alternative for the treatment of bladder cancer.

## INTRODUCTION

Bladder cancer represents one of the most common malignancies worldwide, with approximately 58,950 new cases and 11,820 deaths in the United State in 2015 [1]. Once bladder cancer becomes a muscle-invasive disease (MIBC), the risks for recurrence, progression and mortality will increase dramatically. For localized MIBC, radical cystectomy is routinely performed but the post-operative 5-year survival rate is only about 50% [2], and new and more effective therapeutic strategies are urgently needed.

Oleanolic acid (OA; 3β-hydroxyolean-12-en-28-oic acid), a pentacyclic triterpene compound, has been isolated from more than 1620 plant species and has also been biosynthesized from lupane [3]. OA possesses a wide range of pharmacological properties, such as hepato-protective, anti-diabetic, anti-viral and anti-cancer effects [4], and it has been shown to be effective for the treatment of a variety of human cancers, such as breast cancer [5], gallbladder cancer [6], hepatocellular carcinoma, among others [7]. Our previous study demonstrated that OA effectively inhibited cell viability and proliferation and promoted cell apoptosis and cell cycle arrest at G0/G1 phase via PI3K/AKT pathway in human prostate cancer cells [8]. While an inhibitory effect on the proliferation of T24 cells was suggested [9], the exact underlying mechanism of OA working on bladder cancer has not been fully understood. Several studies have demonstrated that OA induced autophagy and promoted death pathway in cancer cells [10–13], but it is still unclear if autophagy is involved in OA-induced anti-cancer effect.

Autophagy is a highly conserved self-degrading process extensively found in eukaryotic cells and it begins from the formation of double-membrane vacuoles which sequester aggregate-prone proteins, organelles, or portions of the cytoplasm and infectious agents, and ends when they are delivered to the lysosome [14]. As a context-dependent mechanism, autophagy can not only suppress tumor growth but also promote the survival of tumor cells after pharmacotherapy and other intracellular or extracellular stresses [15]. On the one hand, autophagy plays an important role in maintaining cellular homeostasis, and its abnormality may disrupt cellular integrity and promote carcinogenesis. Autophagy was first found to be involved in tumor suppression since autophagic gene BECN1 bore relationship to several human cancers [16, 17]. On the other hand, suppression of autophagy led to oxidative stress, a well-known cause of cancer initiation and progression [18, 19]. Due to increasing biosynthetic and metabolic demands imposed by deregulated proliferation, cancer cells are more dependent on autophagy [15, 20]. Cancer cells exposed to cytotoxic agents may initiate protective autophagy by activating AMPK, GSK3β, ERK1/2 and eEF2K pathways, and autophagy inhibition may enhance the cytotoxicity of anti-cancer agents [21].

In the present study, we investigated if OA induces cytotoxicity or autophagy in bladder cancer cells, and if autophagy, in turn, works on cytotoxicity. Our results revealed that OA induced both cytotoxicity and autophagy in T24 and EJ cells. Moreover, our study also showed that OA promoted autophagy by triggering phosphorylation of AMPKα Thr172, thereby inhibiting mTOR and activating ULK1. Furthermore, we found that inhibition of autophagy enhanced OA-induced cytotoxicity, and on the contrary, induction of autophagy abolished such effects. To sum up, our data suggested that autophagy exerts a cytoprotective effect on bladder cancer cells by compromising OA-mediated anti-cancer effect. These findings indicated that OA in combination with autophagy inhibitors might be a more effective therapeutic alternative for bladder cancer.

## RESULTS

### OA inhibited viability and proliferation of human bladder cancer cells

**Figure 1:**
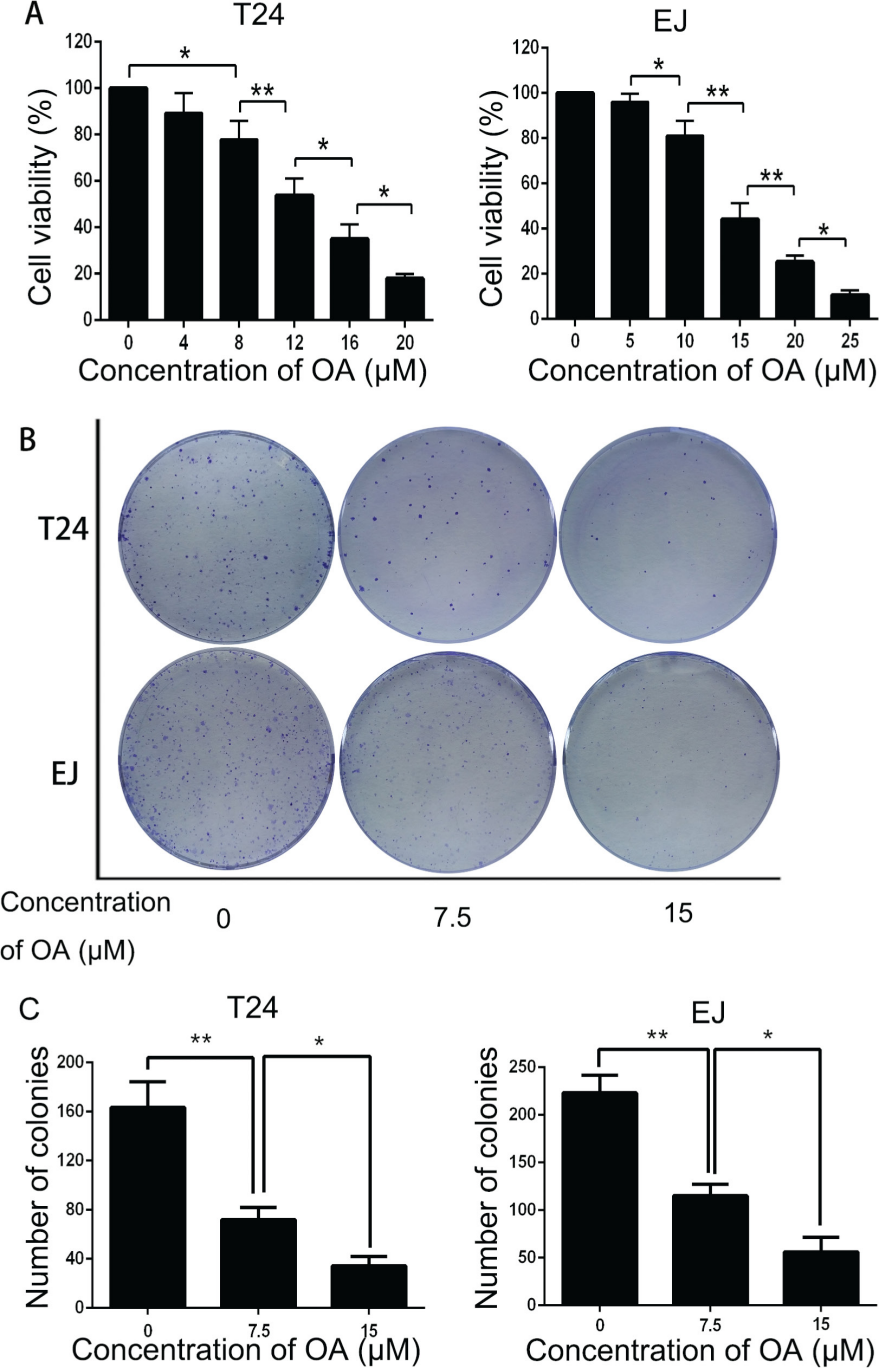
OA inhibited cell viability and proliferation in bladder cancer cells (**A**) Cell viability of T24 and EJ cells. (**B**) and (**C**) Colony formation assay of T24 and EJ cells. The values were represented as mean ± SD of three replicates. *P < 0.05. **P < 0.01.

To determine whether OA inhibits viability and proliferation of human bladder cancer cells, T24 and EJ cells were treated with OA for 24 h. The CCK-8 assay showed that OA treatment decreased cell viability in both cells in a dose-dependent manner (Figure 1A). On the basis of this finding, we chose 7.5 and 15 μM as the appropriate concentration for both cell lines in subsequent experiments. Colony formation assay was applied to evaluate the ability of T24 and EJ cells to form colonies at various different concentrations of OA (Figure 1B) and the results indicated that OA suppressed colony formation dose-dependently in both cell lines (Figure 1C). These findings suggested that OA could inhibit the viability and proliferation of human bladder cancer cells.

### OA induced apoptosis in human bladder cancer cells

**Figure 2:**
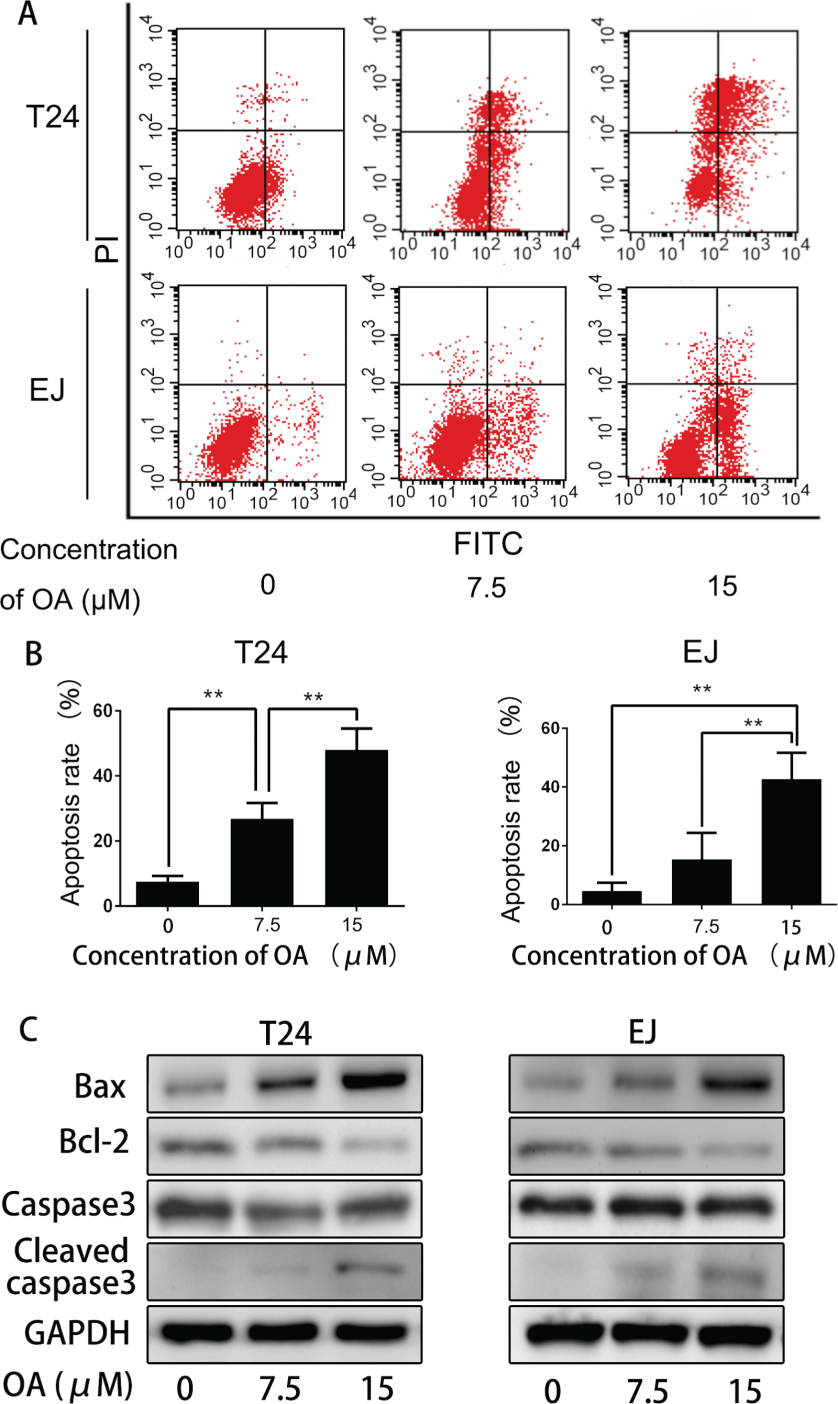
OA induced apoptosis in bladder cancer cells (**A**) and (**B**) Cell apoptosis was measured by flow cytometry. The values were expressed as mean ± SD of three independent experiments. (**C**) OA regulated the expression levels of apoptosis-related proteins in bladder cancer cells. Bax, bcl-2, caspase-3 and cleaved caspase-3 levels were detected by using Western blotting. GAPDH was used as a loading control. *P < 0.05. **P < 0.01.

In order to know whether OA promotes apoptosis in human bladder cancer cells, T24 and EJ cells were incubated with or without 7.5 and 15 μM of OA. The cells were then stained with Annexin V/FITC and PI, and then flow cytometrically examined. As shown in Figure 2A and 2B, in both cell lines, OA treatment increased the number of Annexin V-positive cells in a does-dependent manner. To further understand the mechanisms of OA inducing apoptosis, the expression of some apoptosis-associated proteins were detected, and result revealed that OA activated pro-apoptotic Bax and cleaved caspase-3, while reduced expression of anti-apoptotic Bcl-2 (Figure 2C). These results suggested that OA-induced apoptosis in human bladder cancer cells by controlling of apoptosis regulators.

### OA boosted autophagy in human bladder cancer cells

**Figure 3:**
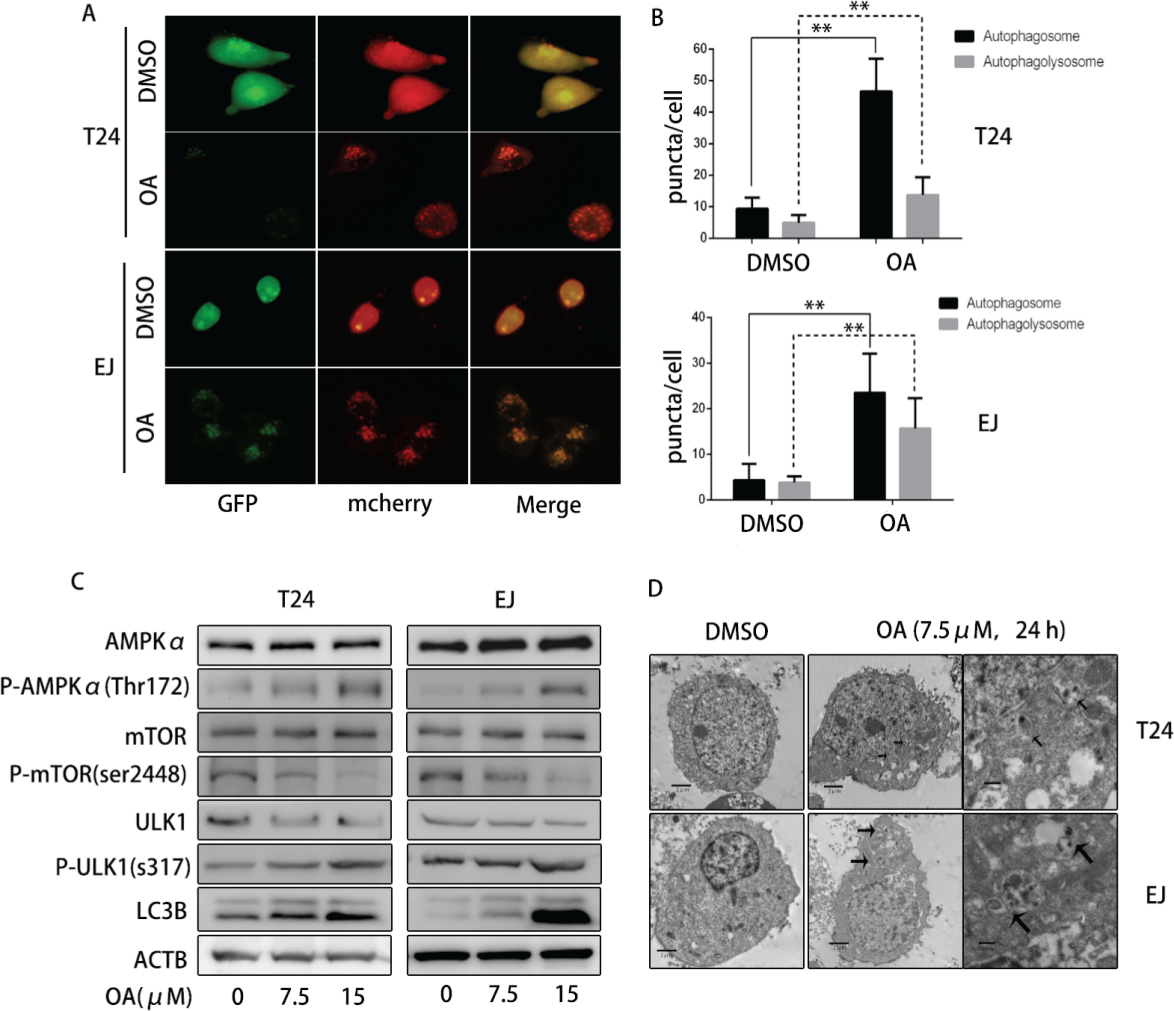
OA induced autophagy through AMPK-mTOR-ULK1 axis in bladder cancer cells (**A**) and (**B**) T24 and EJ pre-transfected with adenovirus mCherry-GFP-LC3B were treated with DMSO (v/v, 1:1000) or OA (7.5 μM) for 24 h, then observed under a fluorescence microscope. (**C**) Expression of AMPKα, P- AMPKα (Thr 172), mTOR, P-mTOR (Ser 2448), ULK-1, P-ULK1 (Ser 317), LC3B was detected by using Western blotting. (**D**) Autophagic vacuoles in T24 and EJ cells were examined under a transmission electron microscope (TEM). *P < 0.05. **P < 0.01.

Since autophagy is an evolutionarily conserved process in response to a variety of stresses including anti-cancer stimuli and plays either a cytopathic or cytoprotective role [22], we investigated whether OA induces autophagy in human bladder cancer cells. First, we transfected cells with adenovirus carrying LC3B (mCherry-GFP-LC3B) for 24 h and then treated the cells with or without OA for another 12 h. Under normal growth conditions, both green (GFP) and red (mCherry) fluorescence diffusely distribute predominantly in the nucleus and cytoplasm. When cells were exposed to various autophagic stimuli, Staining of LC3B with these two fluorescent proteins showed that autophagosome membranes contained LC3B puncta (yellow fluorescentce). Autophagolysosomes displayed only red fluorescent signal (mCherry), which is relatively stable, while GFP fluorescence quenched under the acidic conditions of the lysosome lumen. After treatment with 7.5 μM of OA, T24 and EJ cells were observed under a fluorescence microscope and images were processed with cellSens Entry. Figure 3A and 3B shows that, after OA treatment, the number of yellow puncta (both red- and green-stained, R+G+) and red puncta (red- but not green-stained, R+G−) increased remarkably as compared with the negative control groups. Moreover, we also examined LC3B-II, the lipidized form of LC3B modified from LC3B-I by Western blotting and results showed that LC3B-II level increased gradually in both T24 and EJ cells in a dose-dependent fashion after OA treatment (Figure 3C). These results were further verified by transmission electron microscopy (TEM), which revealed that there existed electron lucent vacuoles in bladder cancer cells following 7.5 μM OA treatments. Finally, autophagosomes were distinctly different from empty vacuoles in that, under higher magnifications, autophagosomes had a double-membrane containing cellular materials and highly electron-dense lysosomal structures (Figure 3D). These findings collectively proved that OA could induce autophagy in human bladder cancer cells.

### OA activated AMPK-mTOR-ULK1 axis in human bladder cancer cells

AMPK is a cellular energy sensor that can be triggered by nutrient starvation or other naturally occurring compounds, thus promoting autophagy [23–25]. To further determine the molecular mechanisms of OA inducing autophagy, the levels of AMPKα and P-AMPKα Thr172 were determined. As shown in Figure 3C, OA treatment did not significantly alter the protein level of total AMPKα, but increased P-AMPKα Thr172 in both T24 and EJ cells. These data indicated that OA might activate AMPK pathway in human bladder cancer cells. Previous study showed that AMPK could suppress mTOR and directly activate ULK1 by phosphorylating ULK1 at Ser317, and finally enhanced autophagy [26]. We then examined several downstream targets of AMPK which are correlated to autophagy such as mTOR, p-mTOR Ser2448 and p-ULK1 Ser317 and the results exhibited that OA treatment did not change the protein level of total mTOR, but appreciably reduced expression of p-mTOR Ser2448 and increased p-ULK1 at Ser317. As a result, inhibition of AMPK reduced the level of p-ULK1 at Ser317 and rescued p-mTOR Ser2448 upon OA treatment. These results indicated that OA might induce autophagy through activating AMPK-mTOR-ULK1 signaling pathway in human bladder cancer cells.

### Blocking autophagy enhanced OA-induced apoptosis in T24 cells

**Figure 4:**
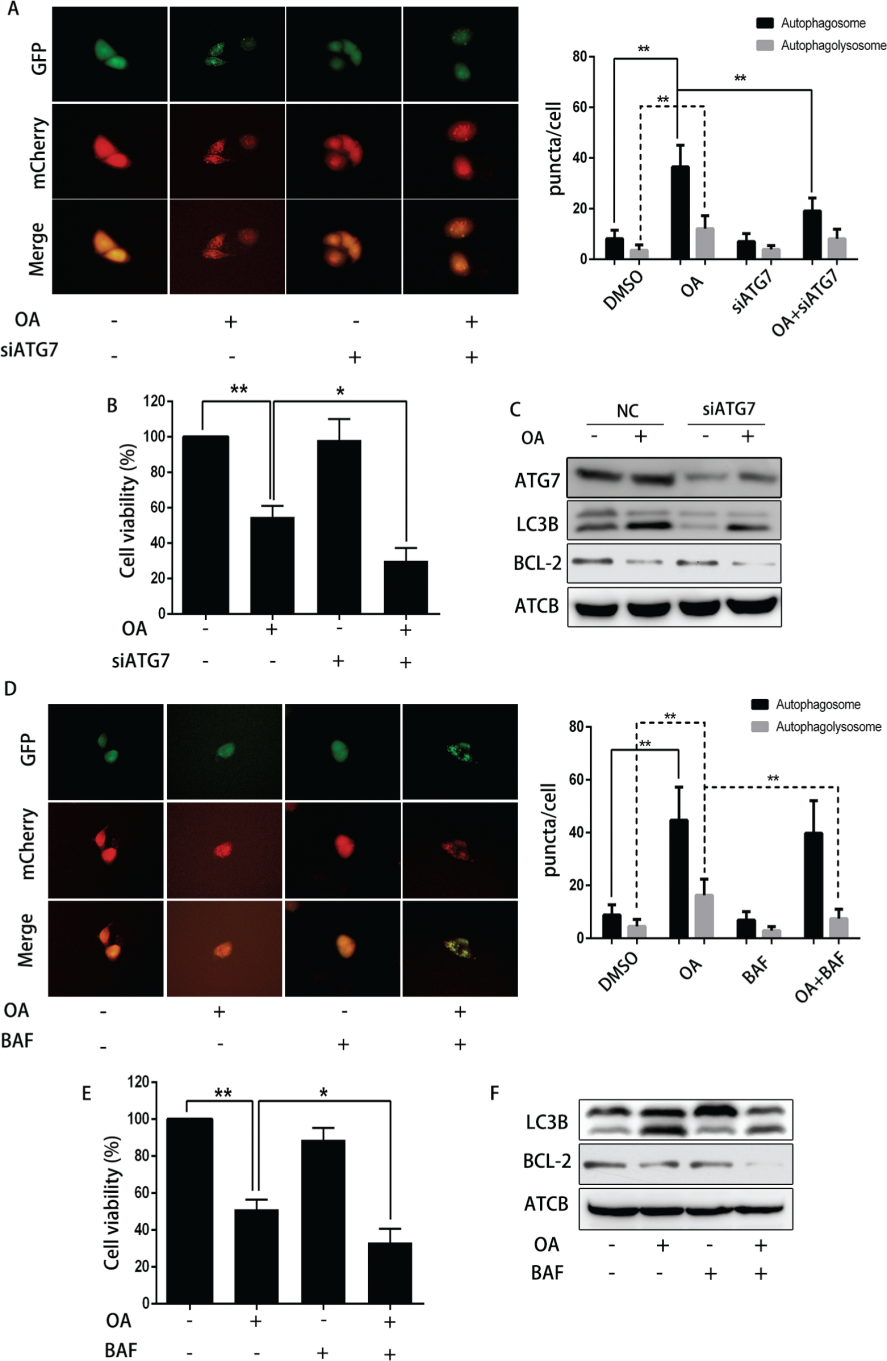
The impact of siRNA ATG7 or bafilomycin A1 on OA-induced anti-cancer effect (**A**) T24 cells pre-transfected with adenovirus mCherry-GFP-LC3B were transfected with siRNA ATG7 or control siRNA for 72 h, then incubated with DMSO (v/v, 1:1000) or OA (7.5 μM) for another 24 h. Cells were then imaged by fluorescence microscopy. (**B**) CCK8 assay was used to analyze the viability of T24 cells. (**C**) Western blotting was used to analyze the protein level of ATG7, LC3B and Bcl-2. (**D**) T24 cells pre-transfected with adenovirus mCherry-GFP-LC3B were pre-treated with bafilomycin (100 nM) for 4 h, then incubated with DMSO (v/v, 1:1000) or OA (7.5 μM) for another 24 h. Cells were then imaged by fluorescence microscopy. (**E**) CCK8 assay was used to analyze the cell viability. (**F**) Western blotting was used to analyze the protein level of LC3B and Bcl-2. **P < 0.05. **P < 0.01.

**Figure 5:**
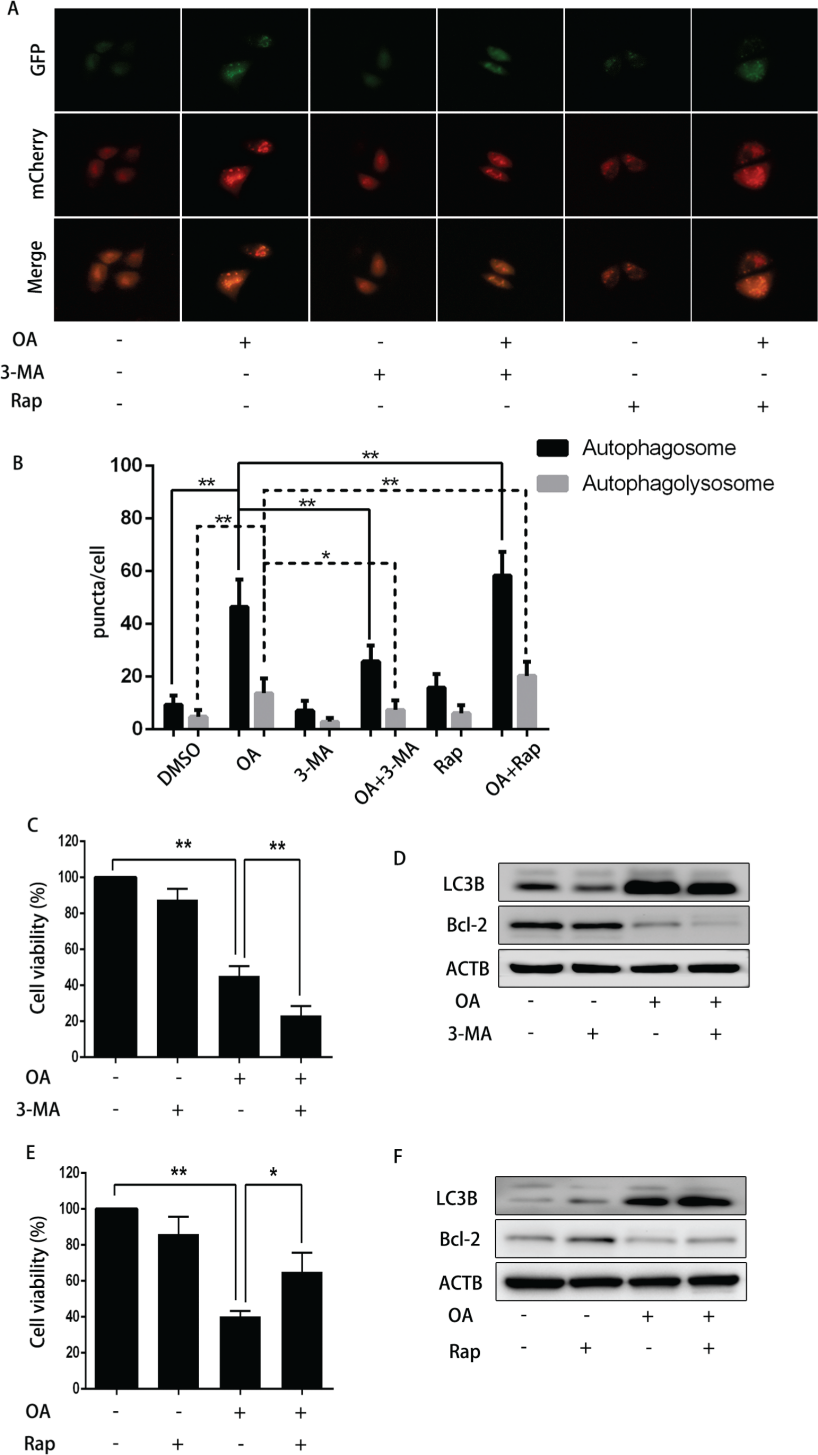
The impact of 3-MA or rapamycin on OA-induced anti-cancer effect (**A**) and (**B**) T24 cells pre-transfected with adenovirus mCherry-GFP-LC3B were pre-treated with 3-MA (5 mM) or rapamycin (200 nM) for 2 h, then incubated with DMSO (v/v, 1:1000) or OA (7.5 μM) for another 24 h. Cells were then imaged by fluorescence microscopy. (**C**) T24 cells pre-treated with or without 3-MA (5 mM) for 2 h were treated with DMSO (v/v, 1:1000) or OA (7.5 μM) for another 24 h. CCK8 assay was used to analyze the cell viability. (**D**) Western blotting was used to analyze the protein level of LC3B and Bcl-2. (**E**) T24 cells pre-treated with or without rapamycin (200 nM) for 2 h were treated with DMSO (v/v, 1:1000) or OA (7.5 μM) for another 24 h. CCK8 assay was used to analyze the cell viability. (**F**) Western blotting was employed to analyze the protein level of LC3B and Bcl-2. **P < 0.05. **P < 0.01.

To verify the effect of autophagy on OA-induced apoptosis, 3-MA (3-Methyladenine, a small molecule inhibitor of autophagy) was used to abolish autophagy in human bladder cancer cells. As shown in Figure 5A and 5B, 3-MA reduced the amount of both yellow puncta (autophagosomes) and red puncta (autophagolysosomes), indicating that 3-MA inhibited the formation of OA-induced autophagosomes. Cell viability was reduced synergistically when treated by OA plus 3-MA as compared treatment with one agent alone in T24 cells (Figure 5C). In line with this finding, Western blotting revealed that 3-MA diminished OA-induced LC3B-II expression. At the same time, 3-MA reduced the expression of anti-apoptotic protein Bcl-2 (Figure 5D). Furthermore, inhibition of autophagy with ATG7 siRNA and bafilomycin A1 also confirmed that blocking autophagy enhanced OA-induced apoptosis of bladder cancer cells (Figure 4). In general, our evidence supported the hypothesis that autophagy may compromise OA-induced cytotoxicity in human bladder cancer cells.

### Activating autophagy compromised OA-induced apoptosis of T24 cells

To further validate our conclusion, we evaluated the effect of another autophagy promoter rapamycin (a specific mTOR inhibitor that induces autophagy) on OA-induced apoptosis. As expected, rapamycin increased the number of both yellow and red puncta, indicating that rapamycin could promote OA-induced autophagosomes formation (Figure 5A and 5B). Accordingly, rapamycin up-regulated OA-mediated LC3B-II formation, and increased cell viability dramatically in the absence of OA. Additionally, rapamycin, by enhancing autophagy, increased expression of Bcl-2 (Figure 5E and 5F). These findings further confirmed that activation of autophagy compromised OA-induced cytotoxicity in human bladder cancer cells.

### AMPK was implicated in protective autophagy upon OA-induced apoptosis in T24 cells

**Figure 6:**
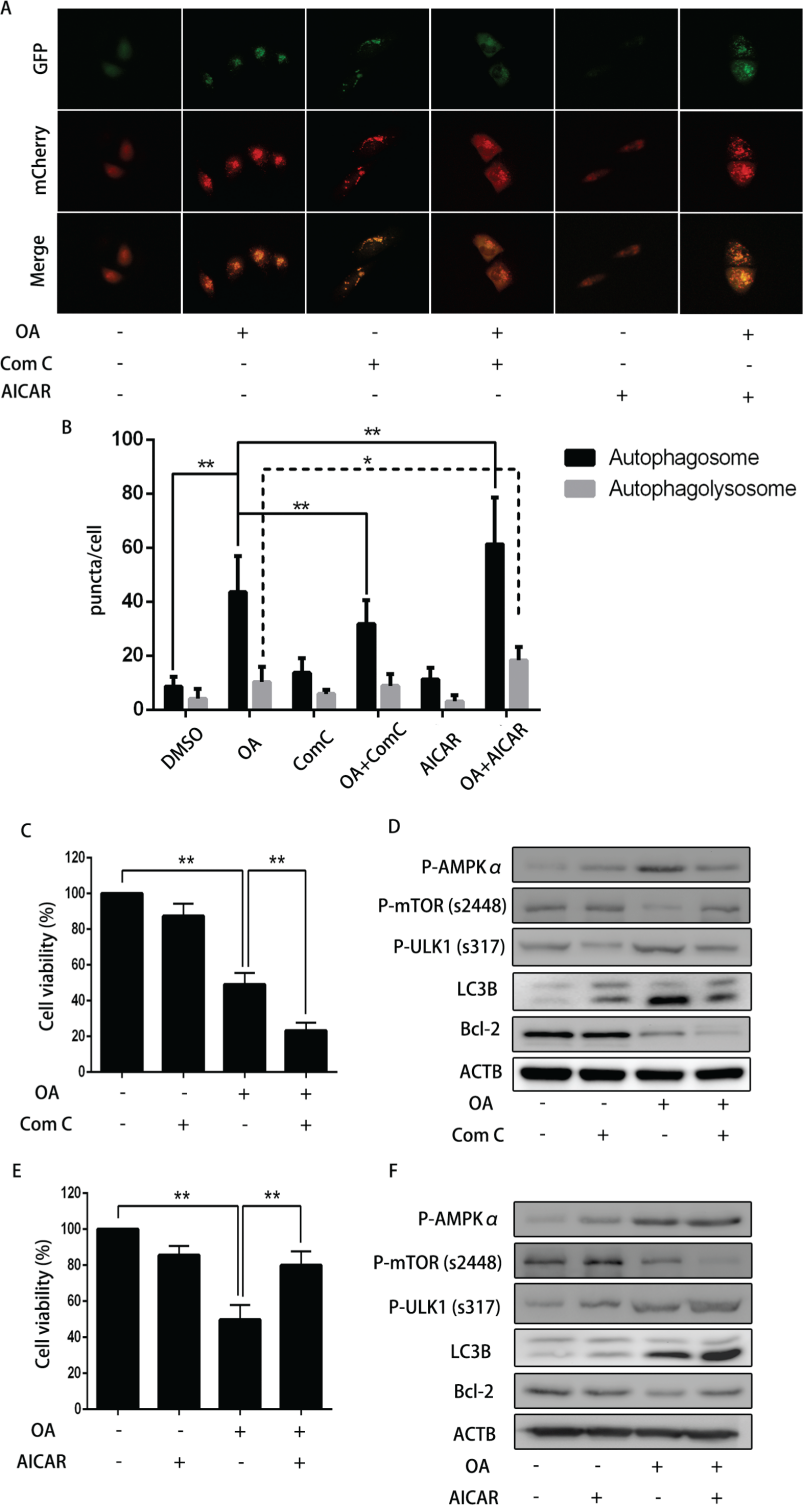
The impact of dorsomorphin or AICAR on OA-induced anti-cancer effect (**A**) and (**B**) T24 cells pre-transfected with adenovirus mCherry-GFP-LC3B (10 MOI) for 24 h were pre-treated with dorsomorphin (10 μM) or AICAR (400 μM) for 2 h, and then incubated with DMSO (v/v, 1:1000) or OA (7.5 μM) for another 24 h. Cells were then imaged by fluorescence microscopy. **P < 0.05. **P < 0.01. (**C**) T24 cells pre-treated with or without dorsomorphin (10 μM) for 2 h were treated with DMSO (v/v, 1:1000) or OA (7.5 μM) for another 24 h. CCK8 assay were used to analyzed the cell viability. (**D**) Western blotting was used to analyze the protein level of LC3B and Bcl-2. (**E**) T24 cells pre-treated with or without AICAR (400 μM) for 2 h were treated with DMSO (v/v, 1:1000) or OA (7.5 μM) for another 24 h. CCK8 assay was used to analyze the cell viability. (**F**) Western blotting was used to analyze the protein level of LC3B and Bcl-2.

To examine the effect of AMPK on OA-induced apoptosis, we employed dorsomorphin, an AMPK inhibitor to abolish OA-induced autophagy in bladder cancer cells. Our results showed that dorsomorphin substantially increased the number of both yellow and red puncta as compared with negative control group. Nonetheless, it decreased the number of both yellow and red puncta in those cells upon treatment with OA (Figure 6A and 6B). Dorsomorphin distinctly inhibited cell viability in the absence of OA (Figure 6C). As shown in Figure 6D, dorsomorphin down-regulated P-AMPKα, P-ULK1 (s317), rescued the level of P-mTOR (s2448).

What is more, dorsomorphin up-regulated LC3B-II expression when compared with the negative control, while it attenuated OA-mediated increment of LC3B-II level. Even though dorsomorphin induced autophagy in T24 cells without OA treatment, it might enhance cytotoxicity by inhibiting AMPK. Then, we used AICAR to activate the AMPK to see if it exerts the similar effect, like an autophagy activator, and our results showed that AICAR increased the amount of yellow and red puncta and LC3B-II formation without OA treatment (Figure 6A and 6B), and AICAR reversed OA-induced inhibitory effect on cell viability in T24 cells (Figure 6E). Western blotting showed that AICAR promoted autophagy in the absence of OA by activating AMPK (Figure 6F).

## DISCUSSION

Previous studies have demonstrated that OA triggered a wide array of biological processes in cancer cells, such as apoptosis and G_0_/G_1_ phase arrest [8]. This study demonstrated that OA could inhibit cell viability/proliferation and induce apoptosis/autophagy in human bladder cancer cells. It is worthy mentioning that OA-induced autophagy *per se* compromised its cytotoxicity, and autophagy inhibitors, like 3-MA and bafilomycin A1, enhanced OA-mediated anti-cancer effect.

Inducing apoptosis has been used as an important strategy for cancer therapy. In current study, OA was found to be capable of inhibiting cell viability and proliferation, and, moreover, OA increased apoptosis in both T24 and EJ cells. Bax and Bcl-2 are crucial apoptotic regulators, and when Bax is in excess of Bcl-2, the ensuring formation of homodimers (Bax–Bax) can promote apoptosis [27]. Cleaved caspase-3 is the mutual downstream effector of many apoptosis pathways, and acts as an executioner protease during apoptosis [28]. Our study showed that upon the administration of higher concentration OA, the protein level of Bax was increased, whereas Bcl-2 declined. After treatment with OA for 24 h, OA cleaved apoptosis marker caspase-3 in both cell lines. These data suggested that OA might induce apoptosis in bladder cancer cells through the mitochondrial pathway.

It has been reported that OA induced apoptosis and autophagy in a variety of cancer cells. OA could recruit LC3B-II formation [10–13], but the crosstalk between apoptosis and autophagy remains to be further clarified. Autophagy, an eukaryotic conserved catabolic process, has been deemed as a prerequisite for cancer development and potential target of cancer treatment due to its involvement with damnification, tumorigenesis and metabolic stress [29, 30]. Multiple natural compounds, such as bergapten and ginsenoside Rh2, can induce autophagy in breast or hepatocellular carcinoma cells [31, 32]. It is known that LC3I converts to LC3II during the formation of double-membraned autophagosomes. Therefore, LC3II is generally considered as a marker of autophagy. Our present study showed that OA treatment conspicuously increased protein level of LC3B-II. Besides, in bladder cancer cells transfected with adenovirus containing LC3B, the number of autophagosomes and autophagolysosomes was increased significantly following 24 h-treatment with OA. Transmission electron microscopy further verified the existence of autophagosomes in both T24 and EJ cells after treatement with 7.5 μM of OA. All these findings suggested that OA might induce autophagy in human bladder cancer cells. Together with several other studies demonstrating that OA induced autophagy in hepatocellular carcinoma and gastric cancer cells [11, 12], OA might be able to induce autophagy in most tumor cells. AMPK is a cellular energy sensor conserved in all eukaryotic cells, and activation of AMPK can lead to dephosphorylation of mTOR and thereby enhance autophagy flux [33]. Our results exhibited that OA suppressed p-mTOR (Ser2448) and increased the levels of p-AMPKα (Thr172) and p-ULK1 (Ser317), indicating that AMPK-mTOR-ULK1 pathway might take part in OA-induced autophagy in bladder cancer cells.

Autophagy is a double-edged sword for the pharmacotherapy of malignancies. Some agents induce autophagic cell death, while others ellicit autophagy which work against their anti-cancer effects. For instance, bergapten [31], norcantharidin [34], hernandezine [35] have been reported to cause autophagic cell death, while berberine [36], 3,4,4-trihydroxy-trans-stilbene [37] induced autophagy as a protective mechanism against the anti-cancer agents. In this study, 3-MA, bafilomycin A1 and ATG7 siRNA were used to block OA- induced autophagy in T24 cells, and the results demonstrated that inhibition of autophagy significantly reduced cell viability. In contrast, rapamycin or AMPK activator AICAR markedly diminished OA-induced cell death.

To sum up, this study demonstrated that OA induced protective autophagy by activating AMPK-mTOR-ULK1 pathway in bladder cancer cells, and combined use of autophagy inhibitors could enhance OA-induced anti-cancer effects. Our findings showed that OA in combination with autophagy inhibitors might be a novel alternative for the treatment of bladder cancer.

## MATERIALS AND METHODS

### Cell culture

T24 and EJ cell lines were cultured in Dulbecco's modified eagle medium (Gibco, Thermo Fisher Scientific Inc., Waltham, MA, USA) supplemented with 10% fetal bovine serum (Gibco) in a 5% CO_2_ humidified incubator at 37°C.

### Reagents and antibodies

Oleanolic acid (OA) was purchased from Sigma-Aldrich (Saint Louis, MO, USA), and was dissolved in dimethyl sulfoxide (Sigma-Aldrich, Saint Louis, MO, USA) at a stock concentration of 30 mM and stored at −20°C. 3-methyladenine (S2767), rapamycin (S1039) dorsomorphin (compound C, S7306) and AICAR (Acadesine, S1802) were bought from Selleckchem (Huston, TX, USA). Bafolimycin A1 were procured from InvivoGen (San Diego, CA, USA). Cell Counting Kit-8 was from Dojindo (Kumamoto, Japan). FITC Annexin V Apoptosis Detection Kit I came from BD biosciences (#556547, Franklin Lakes, NJ, USA). ATG7 siRNA I (6604S) and control siRNA (6568S) were products of Cell Signaling Technology (Beverly, MA, USA). Antibodies against mTOR (6308), phosphor-mTOR (Ser2448) (3308), caspase 3 (6311), cleaved-caspase 3 (7022) were all from Affinity Biosciences (Cincinnati, OH, USA). Antibodies against AMPKα (5831S), phospho-AMPKα (Thr172) (2535S), ULK1 (8084S), phospho-ULK1 (Ser317) (12753S), LC3B (3868S) were bought from Cell Signaling Technology. Antibodies against Bax (23931-1-AP), BCL2 (12789-1-AP), GAPDH antibody (10494-1-AP), β-actin antibody (20536-1-AP), AP conjugated goat anti-mouse and anti-rabbit secondary antibodies were from Proteintech (Chicago, IL, USA).

### Cell viability assay

Cells (5000 cells per well) were plated into 96-well plate, cultured with completed medium, then treated with various dosages of OA or other reagents. 24 h after treatment, 10 μL CCK-8 solution was added to each well and the plate was incubated for 2 h at 37°C. The absorbance at 450 nm was measured on a microplate reader (Tecan, Mannedorf, Switzerland).

### Flow cytometry

Cell apoptosis was detected using the FITC Annexin V Apoptosis Detection Kit I by following the manufacturer's protocols. Cells (5 × 10^5^ per well) were seeded into 6-well plate overnight and then treated with the indicated concentrations of OA for 24 h. Cells were collected, suspended in Annexin V binding buffer, incubated with FITC Annexin V and propidium iodide (PI) and analyzed on a FACSVerse™ flow cytometer (BD Biosciences, San Jose, CA, USA).

### Colony formation assay

Cells (1000 cells per well) were seeded into 6-well plate into 2-ml culture medium overnight. The medium was then changed with fresh medium containing OA (7.5 and 15 μM) or vehicle (DMSO) for 2 days. Subsequently, the medium was changed with fresh medium every 2 days for another 10 days. Colonies were fixed with 4% paraformaldehyde and stained with 0.1% crystal violet for 10 min respectively at room temperature. Colonies consisting of more than 50 cells were counted.

### Tandem mCherry-GFP fluorescence microscopy

Cells (1 × 10^4^ cells per well) were seeded into 12-well plate in 1 ml culture medium overnight, then transfected with adenovirus mCherry-GFP-LC3B (10 MOI) for 24 hours. Cells were treated with or without OA (7.5 μM) for another 24 h. After treatment, the cells were washed with PBS three times and examined under an Olympus IX73 fluorescence microscope (Olympus, Japan). The numbers of GFP and mCherry dots were determined by fluorescent puncta then images were processed with cellSens Entry (Olympus). A total of more than 20 cells under each condition were counted for quantification of autophagy.

### RNA interference of ATG7

T24 cells were seeded into 6-well plates. The next day, when the cells reached a 40% confluency, 100 nM ATG7 siRNA I or control siRNA and 5 μl Lipfectamine 2000 transfection reagent (Invitrogen, Carlsbad, CA, USA) were diluted with 250 μl opti-MEM reduced-serum medium (Gibco, Grand Island, NY, USA) respectively. The medium was incubated for 5 min at room temperature. Afterwards, diluted si-RNAs were added into diluted Lipofectamine 2000 and incubated for 20 min at room temperature. 500 μl of the mixture was added into each well. The medium was replaced with fresh medium after 6 hours.

### Western blotting

Cells were lysed in RIPA buffer containing phenylmethanesulfonyl fluoride and phosphatase inhibitor. The concentration of the protein samples was analyzed by using BCA Protein Assay Kit (Beyotime, Shanghai, China). Equal amounts of proteins from each sample were separated by SDS-PAGE gels ranging from 8% to 15% and electrotransferred onto PVDF membranes (Merck Millipore, Billerica, MA, USA). After incubation with primary and secondary antibodies, the immunoblots were visualized by ECL solution on a BioSpectrum Imaging System (UVP, Upland, CA, USA). GAPDH and β-actin were used as the loading controls. The intensities of the bands were analyzed by using ImageJ.

### Transmission electron microscopy

T24 and EJ cells were treated with or without 7.5 μM of OA for 24 h following fixation with a solution containing 3% glutaraldehyde plus 2% paraformaldehyde in 0.1 mol/L cacodylate buffer (pH 7.4) at room temperature for 2 h. The samples were then post-fixed, dehydrated, embedded, sectioned and double-stained with uranyl acetate and lead citrate, and electron micrographs were taken by employing a Trans-mission Electron Microscope (TECNAI G2 20 TWIN, FEI, Hillsboro, USA).

### Statistical analysis

All experiments were performed at least three times. Data were presented as the mean ± standard deviation (SD). Differences between experimental groups were analyzed by ultilizing one-way or two-way ANOVA. A *P* value < 0.05 was considered to be statistically significant.

## Abbreviations

OA: oleanolic acid
AMPK: AMP-activated protein kinase
mTOR: mechanistic target of rapamycin
ULK1: unc-51 like autophagy activating kinase 1
ATG7: autophagy related 7
3-MA: 3-methyladenine
AICAR: acadesine
MIBC: muscle-invasive bladder cancer
BECN1: beclin1
PI3K: phosphatidylinositol 3-kinase
AKT: serine/threonine kinase 1
GSK3β: glycogen synthase kinase 3 beta
ERK1/2: mitogen-activated protein kinase
eEF2K: eukaryotic elongation factor 2 kinase
BAX: BCL2 associated X, apoptosis regulator
BCL-2: B cell leukemia/lymphoma 2
TEM: transmission electron microscopy
LC3B: microtubule associated protein 1 light chain 3 beta
